# Burnout in Health Professionals According to Their Self-Esteem, Social Support and Empathy Profile

**DOI:** 10.3389/fpsyg.2018.00424

**Published:** 2018-04-20

**Authors:** María del Mar Molero Jurado, María del Carmen Pérez-Fuentes, José Jesús Gázquez Linares, Ana Belén Barragán Martín

**Affiliations:** ^1^Department of Psychology, University of Almería, Almería, Spain; ^2^Department of Psychology, Universidad Autónoma de Chile, Santiago, Chile

**Keywords:** burnout, professional, empathy, healthcare, self-esteem, social support

## Abstract

**Introduction:** Professionals in the healthcare field are in situations that could be a source of stress and sometimes develop burnout syndrome. Self-esteem, social support, and empathy are variables which intervene and influence the appearance of this syndrome.

**Objective:** Identify healthcare professional profiles based on self-esteem, empathy and perceived social support, and analyze the extent to which these profiles show differences in developing burnout.

**Method:** The sample was made up of 719 healthcare professionals with a mean of 38.52 years of age. The Short Questionnaire of Burnout, the Rosenberg Self-Esteem Scale, the Perceived Social Support Questionnaire and the Basic Empathy Scale were used.

**Results:** The results of a cluster analysis with self-esteem, empathy, and perceived social support showed four groups/profiles. Two of them, which included professionals with low self-esteem, differed in the rest of the characteristics. Furthermore, significant differences in burnout scores were found among the groups identified.

**Conclusion:** The results show the need to study burnout with attention to individual and or social characteristics, where self-esteem is shown to be one of the explanatory variables making the main differences among the groups.

## Introduction

Healthcare professionals are exposed to complicated situations that can generate tension they deal directly with persons who suffer from health problems and their families (Fernández-Guzmán et al., 2012). These situations can lead to increased stress and what is known as the burnout syndrome. The number of studies related to this syndrome has grown, because one of the groups where it is most prevalent is healthcare personnel (Navarro et al., 2015).

The burnout syndrome is a psychological and emotional affection associated with work which generates high distress and absenteeism in individuals (Gil-Monte, 2007). At the present time there is no single definition of burnout, although there is a consensus about this syndrome as a response to chronic job stress, which is characterized by the appearance of cognitive impairment, affective wear and negative attitudes and behaviors (Ávila Toscano et al., 2010; Casa et al., 2012). Similarly, emotional exhaustion, depersonalization, and lack of personal accomplishment also contribute to this syndrome (Salillas, 2017).

Burnout is related to sociodemographic variables, such as gender, age or years of professional experience, and so forth (Betancur et al., 2012). With regard to gender, some authors underline its higher prevalence in women than in men (Ballester-Arnal et al., 2016), while others show higher levels of burnout in men than in women (Pera and Serra-Prat, 2002), and finally, Peralta-Ayala and Moya (2017) did not find any gender differences in burnout.

The figures found in research done in recent years on subjects related to burnout differ. This is because the prevalence of burnout is hard to determine, since it depends on the cutoff scores of the scale and/or questionnaire used, as well as the criteria used in each country (Ávila Toscano et al., 2010). For example, the study by Embriaco et al. (2012) found that individuals with depression symptoms showed higher levels of burnout.

Studies determining the prevalence of burnout (Núñez et al., 2010; Barragán et al., 2015) have not only related it with demographic variables, but also with other constructs, such as coping, self-esteem, social identity, social support, empathy, and communication skills. It should be mentioned in this regard that adequate development of communication skills in healthcare professionals acts as a protective factor against the burnout syndrome (García et al., 2013; Leal-Costa et al., 2015).

Similarly, self-efficacy and self-esteem as personal variables are also protectors against the appearance of burnout (Vázquez-Ortiz et al., 2012; Fincka et al., 2018). Both self-esteem and self-efficacy affect the way individuals develop attitudes about themselves, which impacts on their professional development (López et al., 2015). In the relationship of burnout and self-esteem, it has been observed that a lack of personal accomplishment leads to low self-esteem and job demotivation (Sánchez, 2014).

Social identity has a transcendental role in the study of burnout syndrome as a variable which influences both the appearance of social support and assessment of stressful situations (Topa-Cantisano and Morales-Domínguez, 2007). Thus, perceived social support has a mediating role in the response to job stress, but in this case, does so as an organizational protective factor (Topa et al., 2005; Pérez-Fuentes et al., 2014).

Additionally, empathy is a social skill fundamental in developing prosocial behaviors which offer help and favor other persons (Richaud, 2014). Empathy has a cognitive component and another affective one (Hojat et al., 2002), and is a construct composed of four dimensions, adopting perspectives, emotional understanding, empathetic stress, and empathetic joy (López-Pérez et al., 2008). According to a study by Martínez et al. (2015) there is a significant relationship between the dimensions of burnout and the empathy construct. This relationship occurs between emotional exhaustion and empathetic stress on one hand, and between depersonalization and empathetic joy on the other.

From what has been observed up to now, the use of these skills is necessary to be able to manage stress and to manage moods and emotions themselves (Morales, 2017).

Since there are very few studies (Maricutoiu et al., 2017) which show different self-esteem, empathy, and social support profiles of healthcare professionals and the prevalence of burnout in each, the objective posed for this study was, on one hand, to identify the various healthcare professional self-esteem, empathy and perceived social support profiles, and on the other, analyze the extent to which these profiles show differences in burnout.

Based on previous empirical evidence, the following hypotheses were posed: (1) medium/high levels of self-esteem are associated with higher than mean sample empathy and perceived social support, (2) a low level of self-esteem is related to levels below (or similar to) the sample mean in empathy and perceived social support, and (3) there are significant differences in burnout among the groups characterized by medium/high self-esteem and those with low self-esteem.

## Materials and Methods

### Participants

The sample was made up of 719 healthcare professionals. Of these, 11.3% (*n* = 81) were physicians, 7.2% (*n* = 52) where physiotherapists, 52% (*n* = 374) were certified nursing assistants, 6.5% (*n* = 47) were hospital aides and 22.9% (*n* = 165) had other healthcare positions.

Participant age was from 20 to 62 with a mean of 38.52 years (*SD* = 9.45). By gender, 15.7% (*n* = 113) were men with a mean age of 35.33 (*SD* = 8.93), while 84.3% were women, with a mean age of 39.12 (*SD* = 9.44). Participant marital status was 34.8% (*n* = 250) single, 57.1% (*n* = 410) stable partner or married, 0.7% (*n* = 5) widowed, and the rest 4.8% (*n* = 3) were separated or divorced.

### Instruments

An *ad hoc* questionnaire for collecting participant sociodemographic data.

The Short Questionnaire of Burnout (SQB; Moreno et al., 1997) was used to measure burnout. This is a brief instrument for overall evaluation of burnout, as well as syndrome antecedents and consequences. Designed as a questionnaire to supplement the Maslach Burnout Inventory (MBI; Maslach and Jackson, 1986), it consists of 21 items with a five-point Likert-type response scale organized theoretically in three blocks. This study made use of the block of three syndrome factors in the model by Maslach and Jackson (1981). The instrument’s reliability for the study sample for the factor evaluating overall burnout was 0.78 (Cronbach’s alpha).

The Self-Esteem Scale (Rosenberg, 1965) was designed to evaluate how satisfied one feels with oneself. This instrument consists of 10 general items scored from 1 to 4 on a Likert-type scale, where 1 is “strongly agree” and 4 “strongly disagree.” The total score is the result of the sum of the points on the 10 items it consists of, some of which are positive and others are negative, reverse-scored items. The total score on the scale is from 10 to 40 points. Reliability of this study had a Cronbach’s alpha of 0.81 for each scale.

The *Cuestionario de Apoyo Social Percibido* [Perceived Social Support Questionnaire] (CASPE; Calvo and Díaz-Palarea, 2004) consists of nine items which determine whether the subject has a partner and the quality of their relationship (in one item), the relationship with the family in terms of number of contacts and subjective perception of them (in three items), friendships (using four items) and participation in social and cultural organizations (using one item). Items 1–7 are rated on a Likert-type scale with four choices, Item 9 with five choices and one item is answered yes/no. Scoring is done by assigning each item the numerical value of the choice answered (for a possible score of 9 to 35), so that the higher the score, the more perceived social support there is. The Cronbach’s alpha calculated for the instrument from our study sample data was 0.84.

Basic Empathy Scale (BES; Jolliffe and Farrington, 2006). The adaptation by Oliva et al. (2011) was used. It consists of nine items which are distributed in two scales corresponding to Affective Empathy and Cognitive Empathy. These items are answered on a five-point Likert-type scale where 1 = Completely disagree and 5 = Completely agree. Instrument reliability found for the study sample had a Cronbach’s alpha of 0.90 for the affective empathy scale and 0.91 for cognitive empathy.

### Procedure

Participation in this study was voluntary and all the participants who filled in the questionnaire were informed of its objectives and how to fill it in. They were also informed that their answers would be completely anonymous and data processing confidential. The questionnaire was filled in online individually during the months of November 2016 to March 2017. Control questions were included to avoid random answers, and all the participants gave their informed consent to ensure that ethics of research were complied with. Similarly, it should be mentioned that this study was approved by the University of Almería Bioethics Committee.

### Data Analysis

SPSS v23 statistical software was used for data analysis. First a two-step cluster analysis was done to establish the groups of participants based on self-esteem as a categorical variable (low, medium, and high), and other continuous quantitative variables, such as general self-efficacy, empathy (cognitive and affective) and perceived social support.

When the groups or clusters had been identified, an ANOVA was done to determine any significant differences between the groups with respect to burnout as a dependent variable. The Scheffé test for *post hoc* comparisons was used to determine significant differences between means. And the descriptive parameters were found by frequency analysis.

## Results

**FIGURE 1:**
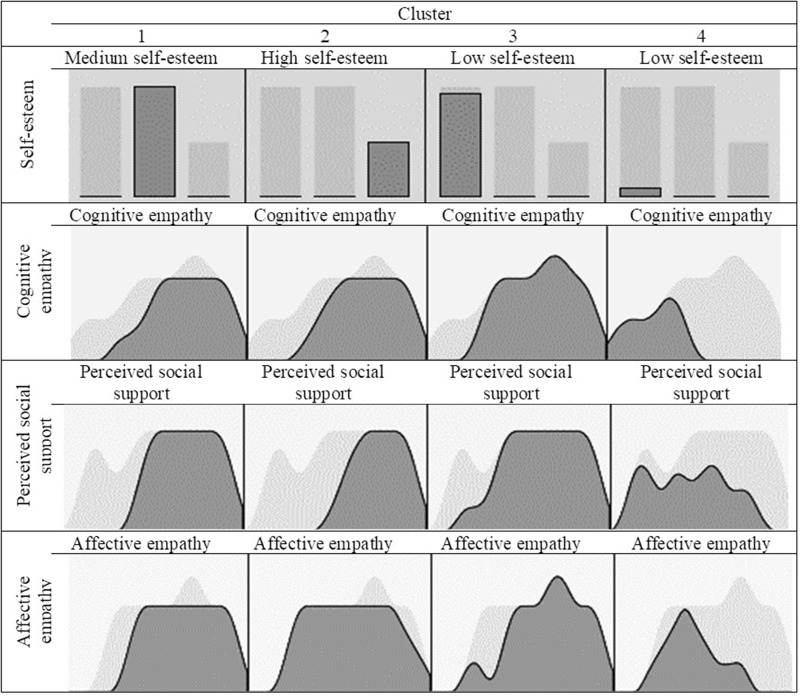
Cluster composition.

A cluster analysis with the following variables was done to form the groups: self-esteem (low, medium, high), cognitive/affective empathy and perceived social support. The four groups resulting from these variables (**Figure 1**) were distributed as follows: 40.1% (*n* = 282) of the participants were in Cluster 1, 19.8% (*n* = 139) in Cluster 2, 37.3% in Cluster 3 (*n* = 262), and the remaining 2.8% (*n* = 20) were in Cluster 4.

As shown in **Table 1**, the first group resulting from the cluster analysis (Cluster 1) was characterized by 100% medium self-esteem and means slightly above those for the total sample in the empathy and social support variables. The specific mean scores in Cluster 1 on each of the variables were cognitive empathy (*M* = 20.08), affective empathy (*M* = 14.76), and perceived social support (*M* = 24.76). Means for the total study sample (*n* = 719) were cognitive empathy (*M* = 19.59), affective empathy (*M* = 14.54), and perceived social support (*M* = 24.20).

**Table 1:** Frequency/ means scores for the total sample and clusters.

	Total sample (*N* = 719)	Cluster			
		1 (*n* = 282)	2 (*n* = 139)	3 (*n* = 262)	4 (*n* = 20)
Low self-esteem	41.3%	–	–	100%	95%
Medium self-esteem	39.4%	100%	–	–	5%
High self-esteem	19.3%	–	100%	–	–
Cognitive empathy	*M* = 19.59 (*SD* = 3.33)	*M* = 20.80 (*SD* = 2.60)	*M* = 20.29 (*SD* = 2.94)	*M* = 19.60 (*SD* = 2.53)	*M* = 7.60 (*SD* = 3.10)
Affective empathy	*M* = 14.54 (*SD* = 3.40)	*M* = 14.76 (*SD* = 3.40)	*M* = 14.53 (*SD* = 3.38)	*M* = 14.94 (*SD* = 2.68)	*M* = 6.35 (*SD* = 3.34)
Perceived social support	*M* = 24.20 (*SD* = 3.55)	*M* = 24.67 (*SD* = 2.79)	*M* = 25.95 (*SD* = 2.66)	*M* = 23.45 (*SD* = 3.17)	*M* = 15.20 (*SD* = 6.47)

The second group (Cluster 2) identified healthcare professionals with high self-esteem (100%), with scores on the cognitive empathy and social support variables above the mean for the total sample, and similar scores on affective empathy. Specifically, mean Cluster 2 scores were (*M* = 20.29) on cognitive empathy (*M* = 14.53) on affective empathy and (*M* = 25.95) on perceived social support.

The third and fourth groups (Clusters 3 and 4) contain the professionals with low self-esteem (100 and 95%, respectively). These two groups are differentiated by their scores on the rest of the variables analyzed. Although their scores were both lower than the total sample in most cases, in Cluster 4, the mean scores were lower than all the rest of the groups and also the total sample: cognitive empathy (*M* = 7.60), affective empathy (*M* = 6.35) and perceived social support (*M* = 15.20). The group of professionals in Cluster 3 had scores on cognitive empathy (*M* = 19.60) and social support (*M* = 23.45) below the mean of the total sample, while for affective empathy (*M* = 14.94) the mean was slightly higher.

The table below summarizes the frequency (low, medium, and high self-esteem) and mean scores (cognitive/affective empathy and perceived social support) of the variables analyzed for the total sample and each of the clusters.

After classifying the groups based on the three-cluster solution, an ANOVA was done to find out the differences in burnout between the clusters followed by the Scheffé test for *post hoc* comparisons.

As observed in **Table 2**, there were significant differences between the clusters (*F*_(3,699)_ = 12.17; *p* < 0.001; $\eta_{p}^{2}$ = 0.05) in burnout scores. The highest mean score in burnout was in Cluster 4 (*M* = 56.38; *SD* = 23.39). However, the *post hoc* comparisons revealed that it is Cluster 3 (*M* = 56; *SD* = 7.98) which shows significant differences in burnout which turns out to be higher than Cluster 2 (*M* = 51.54; *SD* = 6.91) and also Cluster 1 (*M* = 52.59; *SD* = 7.20).

**Table 2:** Means and standard deviations found by groups (Cluster) in burnout, ANOVA and *post hoc*.

	Cluster	*N*	Mean	*SD*	ANOVA		Difference in means
					*F*	Significance	
Burnout	c1	282	52.59	7.20			
	c2	139	51.54	6.91			|c1-c2||c2-c3|^∗∗∗^|c3-c4|
	c3	262	56.00	7.98	12.17	0.000	|c1-c3|^∗∗∗^|c2-c4||c1-c4|
	c4	20	56.38	23.39			

*^∗∗∗^p < 0.001.*

## Discussion and Conclusion

The healthcare field has been found to be conducive to development of the burnout syndrome due to their relations with patients and their families (Fernández-Guzmán et al., 2012). Different healthcare professional profiles have been identified according to their self-esteem, empathy, and social support. Cluster analysis showed the formation of four groups based on self-esteem (low, medium, high), cognitive/affective empathy and perceived social support. The first group showed medium scores in all the variables compared to the total sample. The second group showed high self-esteem, high scores in cognitive empathy and social support and medium scores in affective empathy with respect to the total sample. These results coincide with the study by López et al. (2015) showing that self-esteem affects the attitudes of individuals and their professional performance.

Moreover, the third and fourth groups showed low self-esteem, and were differentiated by their scores on the rest of the variables. In Group 3, the scores were higher with respect to the total sample in cognitive empathy, affective empathy, and social support. Group 4, however, had mean scores lower than the rest of the groups and the total sample. In other words, these two profiles share the same self-esteem characteristic, but differentiate in the rest of the variables. Where Group 4 scored low scores, just as in self-esteem, in Group 3 these variables had high mean scores, and on the contrary, low self-esteem. These results may be due to the number of persons in this profile, since it is rather small compared to Profile 3. In a study by Sánchez (2014), the lack of personal accomplishment led to low self-esteem and demotivation for work.

In addition, the highest scores in cognitive empathy were in Cluster 1, while Cluster 3 scored above the mean in affective empathy, and finally, Cluster 2 had the highest score in social support. These variables are significantly related to the burnout dimensions (Topa-Cantisano and Morales-Domínguez, 2007; Martínez et al., 2015).

It should be mentioned that there were significant differences in burnout results among the four groups, between Group 3 and Group 2, and between Group 1 and Group 3. That is, there were differences in burnout between the group with low self-esteem (Group 3) and the two groups with medium and high scores in self-esteem. These results attract attention, since while Group 4 also had low self-esteem there were no significant differences with the rest of the groups. This could be due to the number of persons in Group 4, which would be one of the limitations of the study. Therefore, future research should use larger study samples.

Finally, it should also be emphasized that the self-esteem variable is what makes the between-group differences in burnout. Therefore, future studies should also make a regression analysis with the self-esteem variable as the explanatory variable to be able to evaluate its weight in burnout.

## Author Contributions

MM, MP-F, and AB: bibliographic review, article writing, data analysis. JG, MP-F, and MM: researchers of the project to which the article data belong.

## Conflict of Interest Statement

The authors declare that the research was conducted in the absence of any commercial or financial relationships that could be construed as a potential conflict of interest.
